# Gut microbial diversity in two insectivorous bats: Insights into the effect of different sampling sources

**DOI:** 10.1002/mbo3.670

**Published:** 2018-07-03

**Authors:** Haonan Wu, Yutong Xing, Haijian Sun, Xiuguang Mao

**Affiliations:** ^1^ Institute of Estuarine and Coastal Research East China Normal University Shanghai China

**Keywords:** 16S rRNA, gut microbiota, *Myotis altarium*, *Rhinolophus sinicus*

## Abstract

The gut microbiota is now known as a key factor in mammalian physiology and health. Our understanding of the gut microbial communities and their effects on ecology and evolution of their hosts is extremely limited in bats which represent the second largest mammalian order. In the current study, gut microbiota of three sampling sources (small intestine, large intestine, and feces) were characterized in two sympatric and insectivorous bats (*Rhinolophus sinicus* and *Myotis altarium*) by high‐throughput sequencing of the V3‐V4 region of the 16S rRNA gene. Combining with published studies, this work reveals that Gammaproteobacteria may be a dominant class in the whole Chiroptera and Fusobacteria is less observed in bats although it has been proven to be dominant in other mammals. Our results reveal that the sampling source influences alpha diversity of the microbial community in both studied species although no significant variations of beta diversity were observed, which support that fecal samples cannot be used as a proxy of the microbiota in other gut regions in wild animals.

## 1 INTRODUCTION

Gut microbiota is often called the “forgotten organ” in its symbiotic host (O’Hara & Shanahan, 2006) and plays essential roles in food digestion, energy harvest, metabolism, and immune training of its host (Donaldson, Lee, & Mazmanian, 2016; Hooper, Littman, & Macpherson, 2012; Hooper, Midtvedt, & Gordon, 2002; Qin et al., 2010; Turnbaugh et al., 2006; Velagapudi et al., 2010). In mammals, large‐scale studies of gut microbiota have been conducted in humans (Huttenhower et al., 2012; Lozupone, Stombaugh, Gordon, Jansson, & Knight, 2012; Saraswati & Sitaraman, 2015; Yatsunenko et al., 2012), mice (Gu et al., 2013; Tanca et al., 2017), other domestic animals such as pigs (Mu, Yang, Su, Zoetendal, & Zhu, 2017) and sheep (Zeng et al., 2017), and to a lesser extent in wild animals, such as bats (Chiroptera). Bats have been largely overlooked in terms of their gut microbiota although they represent the second largest mammalian order and over 20% of mammal species (Simmons, 2005). To date, only eight research papers on the gut microbiota of bats have been published (Banskar, Mourya, & Shouche, 2016; Carrillo‐Araujo et al., 2015; Daniel et al., 2013; Dietrich, Kearney, Seamark, & Markotter, 2017; Galicia, Buenrostro, & García, 2014; Maliničová, Hrehová, Maxinová, Uhrin, & Pristaš, 2017; Phillips et al., 2012; Weinberg et al., 2017). Comparing with a significant number of studies on viruses in bats (Annan et al., 2013; Calisher, Childs, Field, Holmes, & Schountz, 2006; Tong et al., 2013; Yuan et al., 2010), more work is needed to investigate gut bacterial microbiotas and their effects on ecology and evolution of bats.

A majority of previous studies of gut microbiota have focused on fecal samples because they are easily accessible. However, recent studies have shown that microbiota community compositions varied between different gut regions and feces of mice, (Gu et al., 2013; Looft et al., 2014; Mu et al., 2017) and pikas (Li, Chen, et al., 2017) possibly because microbiotas play different functional roles in different intestinal compartments and niches. To date, very few such comparisons have been conducted in wild animals (but see Kohl et al., 2017; Li, Li, et al., 2017) to test whether fecal samples can be used as a proxy of the microbiota in other gut regions.

In the current study, we aim at comparing the composition of the bacterial microbiota of bats from different gut sections. For this aim, we sampled two bat species, *Rhinolophus sinicus* (Rhinolophidae, Yinpterochiroptera) and *Myotis altarium* (Vespertilionidae, Yangochiroptera). Both species live in the same cave. Both of them are insectivorous and feed on insects such as Coleoptera (Hu, Yang, Tan, & Zhang, 2012; Zhang, 1985). Here by focusing on these two bat species, we aim to (a) characterize their core microbiota and compare it to that of other bat species and mammals; (b) compare the microbiota of paired small intestine, large intestine and feces to test whether fecal samples can be used as a proxy of the microbiota in other gut regions.

## 2 MATERIALS AND METHODS

### 2.1 Sample collection and DNA extraction

A total of six adult bats, three males for *Rhinolophus sinicus* (Rhinolophidae, Yinpterochiroptera) and three females for *Myotis altarium* (Vespertilionidae, Yangochiroptera), were captured from the same cave in Yixing city, Jiangsu province, China, in October 2016. Bat sampling procedure was approved by the National Animal Research Authority, East China Normal University (approval ID 20080209). In order to reduce the possibility of contamination, each bat was kept in a single clean and sterile bag. Feces samples were collected in about two hours after bat capturing and transferred to RNase‐free PCR tubes. Then, bats were rapidly euthanized by cervical dislocation to minimize pain and intestinal tissues were taken and transferred to RNase‐free PCR tubes. In this study, intestinal tissues were divided into small intestine (defined as the region close to section of the back stomach, the part of the duodenum) and large intestine (defined as the part of colon, far from the rectum). All samples were immediately frozen in liquid nitrogen in the field and then stored at a −80°C freezer after going back to the laboratory. To harvest the gut microbiota, the intestinal regions were opened and washed at least three times with the physiological saline DEPC (Diethy pyrocarbonate). Here, intestinal samples include collections of luminal contents. Together with fecal samples, a total of 18 samples were generated in the current study (Table 1).

**Table 1:** Number of valid sequences and alpha diversity indices at 32,010 sequences depth in *Rhinolophus sinicus* and *Myotis altarium*. Alpha diversity indices at 7010 sequences depth in *R. sinicus* are shown in bold

Species	Sample sources	Sample ID	Valid sequences	out counts	Chao1	Observed species	Shannon
***R. sinicus***	Small intestine	RS‐1	39115	67	86.43	67 (**49**)	4.77
		RS‐2	39042	65	79.38	58 (**39**)	4.38
		RS‐3	7675	95	**187.17**	**88**	**4.55**
	Large intestine	RL‐1	36358	139	148.25	137 (**129**)	6.45
		RL‐2	37595	116	176.46	107 (**61**)	5.13
		RL‐3	37049	137	174.56	131 (**72**)	4.27
	Feces	RF‐1	34700	95	150.75	93 (**66**)	3.29
		RF‐2	36312	85	110.67	85 (**64**)	4.42
		RF‐3	40176	88	121.00	83 (**63**)	3.57
***M. altarium***	Small intestine	MS‐1	38516	114	132.89	113	5.25
		MS‐2	37819	81	119.11	74	4.69
		MS‐3	32161	116	141.11	116	4.38
	Large intestine	ML‐1	36493	89	100.25	89	5.56
		ML‐2	47840	97	120.50	83	4.13
		ML‐3	37623	76	115.88	72	4.42
	Feces	MF‐1	41447	182	191.00	180	6.63
		MF‐2	47828	158	159.50	157	5.98
		MF‐3	33211	131	147.88	129	2.90

Genomic DNA was extracted from each sample with the QIAamp DNA Stool Mini Kit (Qiagen, Germany). Quantity and quality of DNA were assessed using NanoDrop and agarose gel electrophoresis, respectively. The final DNA was diluted to a concentration of 1 ng/μl and stored at −20°C.

### 2.2 Species identification

To confirm the species identification of bats based on the morphology in the field, we amplified and sequenced the cytochrome b (*cytb*) gene for all individuals. Genomic DNA was extracted from the muscle tissue using DNeasy kits (Qiagen). Details of primers, PCR reaction and the thermal profile for *cytb* have been provided in (Mao, He, Zhang, Rossiter, & Zhang, 2013). Because several genetic lineages have been found for *R. sinicus* in the mainland of China (Mao et al., 2013), we identified the specific lineage for bats used in this study by reconstructing a Maximum Likelihood (ML) tree based on *cytb* sequences of the focal three samples and ones from the previous study. ML tree was reconstructed in the software RAxML 7.2.8 (Berger, Krompass, & Stamatakis, 2011) with GTRGAMMA model and bootstrap supports were estimated from 1,000 replicate searches.

### 2.3 16S rRNA gene amplification and sequencing

The hypervariable V3 and V4 regions of the 16S rRNA gene (456 bp) was amplified with the universal primer pair 343F (5′‐ TACGGRAGGCAGCAG ‐3′) and 798R (5′‐ AGGGTATCTAATCCT‐3′) (Nossa et al., 2010). The PCR mix (25 μl) consists of KAPA HiFi HotStart ReadyMix (2X), 1 μM of each primer and 2.5 μl of DNA. The thermal profile includes an initial denaturation at 95°C for 3 min, followed by 25 cycles of 95°C 30 s, 55°C 30 s and 72°C 30 s, and a final extension at 72°C 10 min. Amplicons were purified with AMPure XP beads (Agencourt) and amplified in duplicate in independent PCRs labeled using different barcodes. After purification, the final amplicon was quantified using Qubit dsDNA assay kit. Equal amounts of purified amplicon from each sample were pooled (∼20 ng per sample) and sequenced using 350 bp paired‐end reads on Illumina MiSeq platform.

### 2.4 Data analysis

Raw reads were filtered using Trimmomatic software (Bolger, Lohse, & Usadel, 2014) with a sliding window of 4:20 and minlen of 50 bp. Filtered reads were merged using FLASH software (Reyon et al., 2012) with 10 bp of minimal overlapping, 200 bp of maximum overlapping and 20% of maximum mismatch rate. Using QIIME software (version 1.8.0) (Caporaso et al., 2010), sequences with ambiguous bases or homopolymers were discarded and only sequences with the length of over 200 bp and 75% of bases over Q20 were retained. Then, sequences with chimera were detected and removed using USEARCH (Edgar, Haas, Clemente, Quince, & Knight, 2011). The final valid sequences were used for the downstream analysis in QIIME.

Valid sequences were grouped into operational taxonomic units (OTUs) (Blaxter et al., 2005) at a 97% similarity threshold using UPARSE (Edgar, 2013) and the most abundant sequence was picked as the OTU representative sequence using QIIME package. OTUs were assigned taxonomic identities in QIIME using RDP classifier (Wang, Garrity, Tiedje, & Cole, 2007) and the Silva database Version 123 (16s rDNA) (Quast et al., 2012).

**Figure 1:**
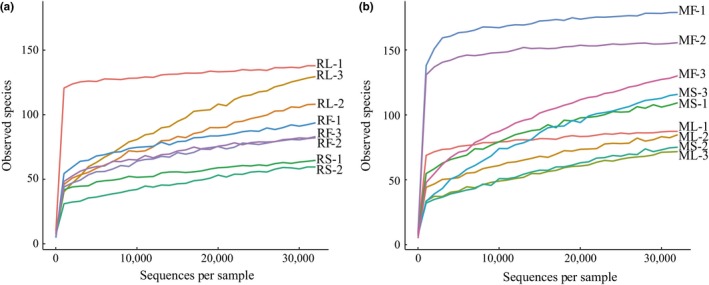
Rarefaction analysis of gut bacteria sequencing of the 16S rRNA gene in different samples of *R. sinicus* and *M. altarium*. (a) Rarefaction curves of eight samples in *R. sinicus* at 32,010 sequences depth. (b) Rarefaction curves of nine samples in *M. altarium* at 32,010 sequences depth

Similar numbers of sequences were generated per sample ranging from 32,161 to 47,840 (Table 1) except for RS‐3 with only 7,675 sequences. To avoid potential artifacts of uneven sequences depth in calculating relevant indices, all samples were rarified to 32,010 sequences except for RS‐3. Rarefaction curves at this sequence depths showed that a majority of microbial diversity was captured for each sample (Figure 1). For comparison with results from 32,010 sequences depth, samples of *R. sinicus* were also analyzed at 7,010 sequences depth (Supporting Information Figure S1).

### 2.5 Effects of sampling sources on the microbial diversity

To determine the effect of sampling sources (the small intestine, large intestine, and feces) on microbial diversity, microbiota composition and abundance were compared among different sampling sources in each of two bat species (*R. sinicus* and *M. altarium*) using both alpha and beta diversity analyses.

The Observed Species counts were used as the indicator of alpha diversity in the samples, but Chao1 and Shannon indices were also generated for comparisons with the published literatures. To compare microbiota abundance difference, genera with the top 15 abundance were obtained and visualized using barplots. The membership and structure of samples at the top 15 abundance genera were revealed by heatmap plots. For comparisons between three sampling sources, one‐way ANOVA was used. For comparisons between two sampling sources (pairwise comparisons), Welch’s *t* test was used. Significant difference was considered at *p* < 0.05.

Beta diversity (between sample diversity) was estimated by calculating unweighted and weighted UniFrac distance matrices (Lozupone, Lladser, Knights, Stombaugh, & Knight, 2011) between samples. The unweighted UniFrac matrix is affected by presence/absence and weighted UniFrac matrix incorporates information of relative abundance. To compare gut community membership and structure, principal coordinates analysis (PCoA) was performed based on unweighted and weighted UniFrac distance matrices. Then, a permutational multivariate analysis of variance was performed based on distance matrices implemented in the adonis function of the vegan package in R. Specifically, we examine the effect of sampling sources on both unweighted and weighted UniFrac distance matrices (beta diversity variation) in each species.

To test the validity of using fecal samples as a proxy of microbiota in other gut regions, we generate UPGMA (Unweighted Pair Group Method with Arithmetic mean) trees based on unweighted and weighted UniFrac distance matrices across all 18 samples.

## 3 RESULTS

### 3.1 Core microbiota in two insectivorous bats

In this study, we amplified and sequenced the cytochrome b (*cytb*) gene for all individuals (GenBank accessions:MH325072‐MH325077). By performing BLAST searches at NCBI database, we confirmed their taxonomic assignments made based on the morphology in the field. In addition, Maximum Likelihood (ML) tree reconstructed based on *cytb* sequences revealed that all three *R. sinicus* samples used in this study were classified with individuals from East *R. s. sinicus* (Supporting Information Figure S2).

**Figure 2:**
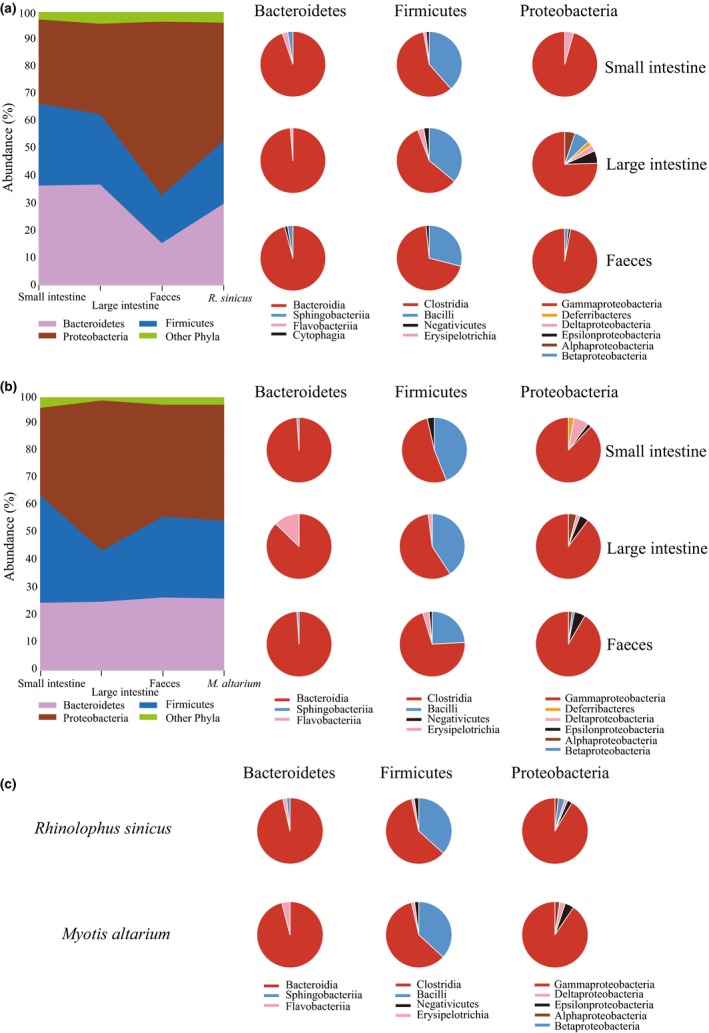
Bacteria community composition and relative abundance at the phyla and class levels in (a) *R. sinicus* and (b) *M. altarium*. (c) Pie charts show relative abundances of bacterial classes with an abundance of > 1% in three dominated phyla in *R. sinicus* and *M. altarium*

All sequences of the 18 samples clustered in 409 OTUs and 403 of them were assigned into order level (L4, 98.5%), 393 into family level (L5, 96.1%), 313 into genus level (L6, 76.5%), and 147 into species level (L7, 35.9%). The dominant phyla in *R. sinicus* and *M. altarium* were Proteobacteria (43.5% in *R. sinicus*; 42.5% in *M. altarium*), Firmicutes (22.9% in *R. sinicus*; 28.7% in *M. altarium*), and Bacteroidetes (29.9% in *R. sinicus*; 26.3% in *M. altarium*), which consistently occurred in all three sampling sources of each species (Figure 2a,b). In the small intestine, the distribution of the three dominant phyla was relatively average in both bat species, whereas in the large intestine of *M. altarium* and feces of *R. sinicus,* Proteobacteria occupied over a half (Figure 2a,b). In *R. sinicus*, other phyla with more than 1% abundance were Acidobacteria (1.0%) in the small intestine and Actinobacteria (2.0%) in the large intestine. In *M. altarium*, other phyla with more than 1% abundance were Actinobacteria (1.72%) and Gemmatimonadetes (1.01%) in the small intestine and Actinobacteria (1.08%) in the feces.

In Proteobacteria, the most abundant class was Gammaproteobacteria (91.4% in *R. sinicus*, 89.9% in *M. altarium*) (Figure 2c) which had a relative abundance of more than 95% in the small intestine and feces and 77.2% in the large intestine of *R. sinicus* and nearly 90% relative abundance in all three sampling sources of *M. altarium* (Figure 2a,b). Other classes of Proteobacteria were Alphaproteobacteria, Betaproteobacteria, Deferribacteres (not observed in the small intestine of *R. sinicus* and large intestine of *M. altarium*), Deltaproteobacteria, and Epsilonproteobacteria (Figure 2a,b). The case of Firmicutes was almost the same between *M. altarium* and *R. sinicus* and the majority classes (96.4% in both species) are Clostridia and Bacilli in all three sampling sources (Figure 2a–c). In Bacteroidetes, the most abundant class (96.2% in *R. sinicus*, 94.9% in *M. altarium*) (Figure 2c) was Bacteroidia in all three sampling sources of *R. sinicus* and the small intestine and feces of *M. altarium* and in the large intestine of *M. altarium* Flavobacteriia class exceeded 12.5% (Figure 2a,b).

Similar numbers of OTUs were observed in the two bat species (318 for *R. sinicus* and 362 for *M. altarium*). This similarity was also reflected at the genus level (108 genera in *R. sinicus* and 117 genera in *M. altarium*). Although a large proportion of taxa was shared between the two bat species (i.e. 96 out of the total 129 genera, 74.4%), 12 unique genera were observed in *R. sinicus* and five of them with an abundance of > 0.1% (*Chryseobacterium*,* Sphingomonas*,* Fusobacterium*,* Moraxella*, and *Ruminococcus 2*), and 21 unique genera were observed in *M. altarium* and six of them with an abundance of > 0.1% (*Campylobacter*,* Flavobacterium*,* Haliscomenobacter*,* Maribacter*,* Neptuniibacter*, and *Ruminococcaceae_UCG_009*). Among 96 shared genera, the most abundant is *Vibrio* which represented 15.5% in *R. sinicus* and 18.1% *M. altarium*.

### 3.2 Effects of sampling sources on the microbial diversity

**Figure 3:**
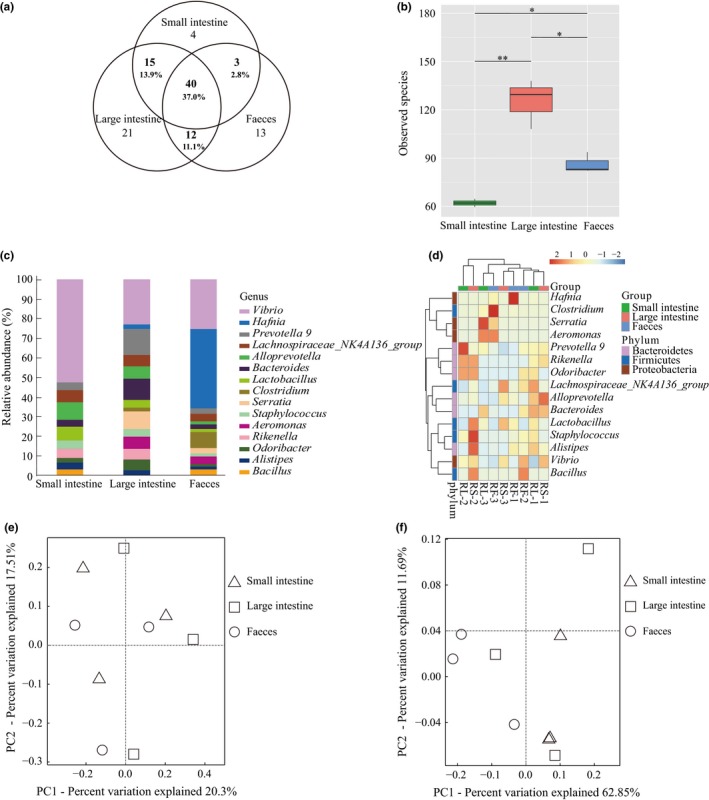
Comparisons of the microbial community composition and abundance in three sampling sources of *R. sinicus*. (a) Venn diagrams of shared genera in three sampling sources. (b) Statistical comparisons of alpha diversity (measured by the total number of observed species) of microbiota among the three sampling sources at 32,010 sequences depths. *p < 0.05, **p < 0.01. (c) Relative abundances of the top 15 genera in three sampling sources. (d) Heatmap of the top 15 abundance genera in three sampling sources. Principal coordinates analysis (PCoA) of all three microbial communities of *R. sinicus* regions based on unweighted UniFrac distances (e) and weighted UniFrac distances (f)

In *R. sinicus*, the large intestine contained the largest number of observed taxa followed by feces (Table 1). Similar pattern was observed in the total and unique number of genera. Specifically, among 108 genera found in all *R. sinicus* samples after removing four uncultured genera, 88 (21 of them are unique) were in large intestine, 68 (13 are unique) in feces, and 62 (four are unique) in small intestine (Figure 3a). The small intestine and feces shared the least number of genera and similar number of genera was shared by large intestine and small intestine and by large intestine and feces (Figure 3a). A total of 40 genera (37.0%) were shared among the three sampling sources (Figure 3a).

At 32,010 sequences depth, a significant difference of alpha diversity (observed species) was detected among the three sampling sources (one‐way ANOVA test, *p* = 0.0038) (Figure 3b). In addition, pairwise comparisons of the three sampling sources were also significant (*p* < 0.05 in all Welch’s *t* test) (Figure 3b). However, at 7,010 sequences depth the difference of alpha diversity among the three sampling sources was not significant (*p* = 0.411) (Supporting Information Figure S3) and also for the three pairwise comparisons (*p* > 0.05 in all Welch’s *t* test). These results indicated that sequences depth generated for each sample might affect alpha diversity measurements. Although we did not recover any OTUs whose abundance differed significantly among the three sampling sources (one‐way ANOVA test, *p* > 0.05), we did find some taxa with a relative high abundance in specific sampling sources. Specifically, among genera with the top 15 abundance small intestine has more *Vibrio*; large intestine has more *Serratia, Prevotella 9,* and *Bacteroides*; the feces have more *Hafnia* and *Clostridium* (Figure 3c). This abundance difference among different sampling sources was largely due to a specific sample and not consistent across all three samples, as revealed by the heatmap (Figure 3d). For example, more *Serratia*,* Prevotella 9*, and *Bacteroides* in large intestine resulted from an extremely high abundance of each genus in RL‐3, RL‐2 and RL‐1, respectively. Similar findings were observed in the high abundance of *Hafnia* and *Clostridium* in feces. However, a high abundance of *Vibrio* observed in small intestine did occur in all three samples. In addition, *Vibrio* was the only genus showing a significant abundance difference among pairwise comparisons (i.e. the small intestine vs. large intestine, *p* = 0.0316, Welch’s *t* test). Overall, heatmap plots of the top 15 abundance genera revealed that samples were mixed and did not cluster based on their corresponding sampling sources (Figure 3d).

Contrast to alpha diversity variation above, sampling sources had no significant effects on beta diversity of both microbial community membership (presence/absence of species; Adonis: *R* ^2^ = 0.245, *p* = 0.523; Figure 3e) and structure (incorporates information of relative abundance; Adonis: *R* ^2^ = 0.461, *p* = 0.083; Figure 3f).

**Figure 4:**
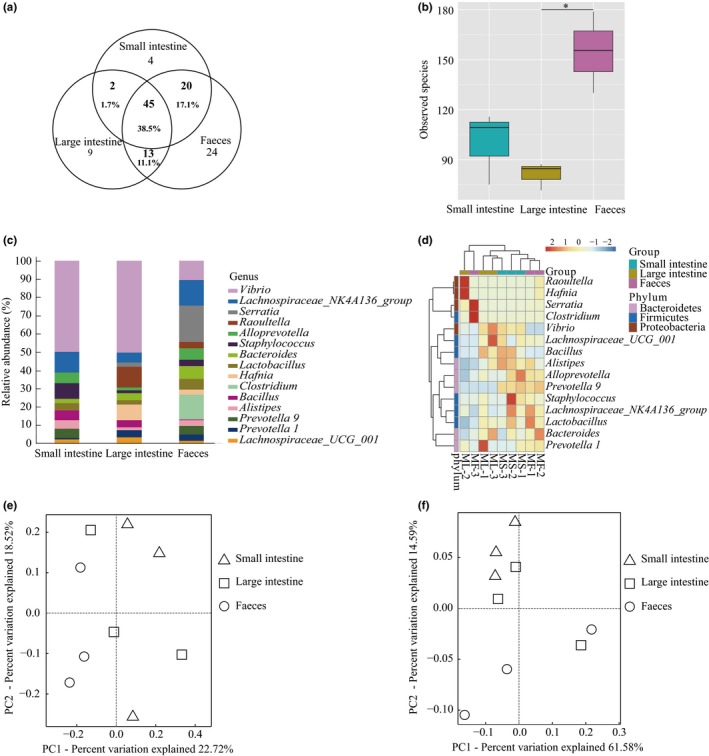
Comparisons of the microbial community composition and abundance in three sampling sources of *M. altarium*. (a) Venn diagrams of shared genera in three sampling sources. (b) Statistical comparisons of alpha diversity (measured by the total number of observed species) of microbiota among the three sampling sources at 32,010 sequences depth. *p < 0.05. (c) Relative abundances of the top 15 genera in three sampling sources. (d) Heatmap of the top 15 abundance genera in three sampling sources. Principal coordinates analysis (PCoA) of three microbial communities of *M. altarium* based on unweighted UniFrac distances (e) and weighted UniFrac distances (f)

In *M. altarium*, feces have the largest number of observed taxa followed by the small intestine (Table 1). Similar to the case in *R. sinicus*, this pattern was also observed in the genus level. Specifically, a total of 117 genera were found in all *M. altarium* samples after removing four uncultured genera, including 102 (24 of them are unique) in feces, 71 (nine are unique) in small intestine, and 69 (four are unique) in large intestine (Figure 4a). Contrast to the results in *R. sinicus*, small intestine and feces shared the most number of genera (20, 17.1%) and only two and 13 genera were shared by large intestine and small intestine and by large intestine and feces, respectively (Figure 4a). A total of 45 genera (38.5%) were shared in three sampling sources (Figure 4a).

Like the case in *R. sinicus*, a significant difference of alpha diversity was observed among the three sampling sources in *M. altarium* (one‐way ANOVA test, *p* = 0.0113) (Figure 4b). However, in pairwise comparisons, significant difference was only found between the large intestine and feces (Welch’s *t* test, *p* = 0.0415) (Figure 4b). For microbiota abundance, five OTUs showed significant differences among the three sampling sources (*p* < 0.05). Among all pairwise comparisons of the top 15 abundance genera, only *Prevotella 9* and *Vibrio* exhibited significant abundance differences (*Vibrio* between small intestine and feces, *p* = 0.00199; *Prevotella 9* between small intestine and large intestine, *p* = 0.0347, Welch’s *t* test) (Figure 4c). Some genera, such as *Raoultella*,* Hafnia*,* Serratia*, and *Clostridium,* showed a high abundance in one sampling source mainly caused by a specific sample and not consistent across all samples (Figure 4d). Unlike the case in *R. sinicus*, samples from the same sampling source clustered together as revealed by heatmap plots of the top 15 abundance genera (Figure 4d).

In line with the results in *R. sinicus*, sampling sources had no significant effects on beta diversity of both microbial community membership (presence/absence of species; Adonis: *R* ^2^ = 0.275, *p* = 0.283; Figure 4e) and structure (incorporates information of relative abundance; Adonis: *R* ^2^ = 0.231, *p* = 0.294; Figure 4f).

**Figure 5:**
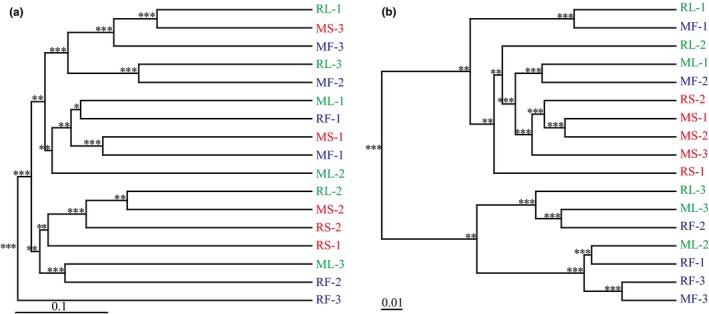
UPGMA clustering analysis of three gut microbial communities of *R. sinicus* and *M. altarium*. (a) Unweighted UniFrac distances. (b) Weighted UniFrac distances. In both trees, samples from the small intestine, large intestine, and feces are coded by red, green, and blue, respectively. Asterisks in the node represent confidence values: *** 75%~100%, ** 50%~75%, * 25%~50%

Finally, the result of UPGMA clustering revealed that fecal community in each species did not cluster with communities of either small or large intestine in terms of both community membership and structure (Figure 5).

## 4 DISCUSSION

### 4.1 **Core** microbiota **in** *R. sinicus* and *M. altarium*

The dominant phyla in *R. sinicus* and *M. altarium* are Proteobacteria, Bacteroidetes and Firmicutes, consistent with the previous study on other insectivorous bats of Phyllostomid (Carrillo‐Araujo et al., 2015). Similar relative abundance of Proteobacteria was observed between our study (>42% in both species) and the previous study on other insectivorous bats (40%, Carrillo‐Araujo et al., 2015). Interestingly, Proteobacteria is rarely found (<10%) in carnivore species except for domestic dogs (Handl, Dowd, Garcia‐Mazcorro, Steiner, & Suchodolski, 2011). Unlike the case of Proteobacteria, the relative abundance of Bacteroidetes (>26%) and Firmicutes (>22%) in *R. sinicus* and *M. altarium* is much higher than other insectivorous bats of Phyllostomid (10% Bacteroidetes and <5% Firmicutes) (Carrillo‐Araujo et al., 2015). Firmicutes is the only phylum universally shared in mammals (Ley et al. 2008) and in some carnivore species and human Firmicutes has a relative abundance of >60% (Menke et al., 2014, 2017).

At the class level, Gammaproteobacteria (Proteobacteria), Bacteroidia (Bacteroidetes), Bacilli (Firmicutes), and Clostridia (Firmicutes) are four dominant classes in *R. sinicus* and *M. altarium*. Gammaproteobacteria is the largest class in *R. sinicus* (39.7%) and *M. altarium* (38.2%), which is higher than other insect‐feeding bats (~20%) (Carrillo‐Araujo et al., 2015). Interestingly, Gammaproteobacteria also has a relatively high abundance in noninsectivorous bats of Phyllostomid (Carrillo‐Araujo et al., 2015). Thus, Gammaproteobacteria may be a dominant class in the whole Chiroptera. In some mammals such as cheetch and vultures feeding on decaying meat, Fusobacteria is dominant at the class level (Menke et al., 2017; Roggenbuck et al., 2014). However, we did not observe Fusobacteria in *R. sinicus* and *M. altarium*, which is also rare in noninsectivorous bats of Phyllostomid (Carrillo‐Araujo et al., 2015).

### 4.2 Effects of sampling sources on microbial diversity

The current study revealed significant differences of the microbial community composition (the alpha diversity) in different sampling sources (the large intestine, small intestine, and feces) in two bat species (*R. sinicus* and *M. altarium*) although no significant variations of beta diversity were observed. These results were in line with the previous studies in mice (Gu et al., 2013; Pang, Vogensen, Nielsen, & Hansen, 2012; Weldon et al., 2015), pigs (Looft et al., 2014; Mu et al., 2017), and sheep (Zeng et al., 2017). Observed differences across the sampling sources may be caused by environmental heterogeneity in different intestinal compartments and niches, such as different oxygen exposure, pH, and substrate availability (Hao & Lee, 2004). In addition, functional changes have also been reported between cecal and fecal microbiota in mouse (Tanca et al., 2017). Our study further confirms that fecal samples, although easily accessible, cannot be used as a proxy of the microbiota in other gut regions (Li, Li, et al., 2017b).

Our current results contrast with the previous work on Phyllostomid bats that did not reveal significant differences in microbiota composition between three different intestine regions (Carrillo‐Araujo et al., 2015). This contrast may be caused by different sampling sources used in comparisons. It is now known that microbiota differ across different functional gut regions (Haange et al., 2012). In Carrillo‐Araujo et al. (2015), the intestine was divided into three fractions of similar size (anterior, medium, and posterior). In addition, fecal samples were not included in Carrillo‐Araujo et al. (2015). Significant differences in microbiota composition between three different sampling sources observed in the current study may result from the inclusion of fecal samples. Indeed, as for the microbiota difference between large and small intestine, our results in *M. altarium* were consistent with Carrillo‐Araujo et al. (2015). In addition, sequences depth per sample may also contribute to the contrast between our study and Carrillo‐Araujo et al. (2015). The current study analysis based on a low sequences depth (7,010 sequences) in *R. sinicus* did not reveal a significant difference of microbial community compositions across the sampling sources.

Although two genera were identified to exhibit significant abundance differences between different sampling sources, they were not consistent in two bat species. For example, *Vibrio* showed a significant abundance difference between small intestine and large intestine in *R. sinicus* but between small intestine and feces in *M. altarium* and a significant abundance difference of *Prevotella* 9 was only observed between small intestine and large intestine in *M. altarium*. Thus, our current results cannot draw any conclusions about the effect of sampling source on microbial community abundance.

## 5 CONCLUSION

This study has characterized the microbiota of three sampling sources (the small intestine, large intestine, and feces) in two insectivorous bat species. Our study adds to the list of a growing number of studies on the gut microbiota in bats. Our results revealed that the sampling source influences the alpha diversity of microbial community and suggest that fecal samples cannot be used as microbial inventories in other gut regions. In the future, more number of individuals will be needed to test this suggestion. Recent studies have shown that the sex of hosts may affect the gut microbial diversity (e.g. Fierer, Hamady, Lauber, & Knight, 2008; Markle et al., 2013). In this study, all *R. sinicus* individuals are male and all *M. altarium* are females. Thus, we did not compare the difference of microbial diversity between these two bat species. Future investigations will continue to assess the relative effects of genetic divergence of hosts, sex, the gut region, and diet on gut microbial communities.

## CONFLICT OF INTEREST

None declared.

## DATA ACCESSIBILITY

All the raw Illumina sequencing reads have been deposited to the NCBI Sequencing Read Archive (SRA) database under the accession ID SRP144939.
