# Supplementary material for: Gut microbial diversity in two insectivorous bats: Insights into the effect of different sampling sources
Source: Microbiologyopen. 2018 Jul 3;8(4):e00670. doi: 10.1002/mbo3.670 (PMC6530527; doi:10.1002/mbo3.670)
Supplement: Supplementary file 1 [file MBO3-8-e00670-s001.docx]

**Figure S1** Rarefaction analysis of gut bacteria sequencing of the 16S rRNA gene in different samples of *R. sinicus* at 7010 sequences depth.

**Figure S2** A Maximum Likelihood (ML) tree reconstructed based on *cytb* sequences of the three *R. sinicus* samples and ones from the previous study (Mao et al. 2013). Red arrows represent the three *R. sinicus* samples used in this study.

**Figure S3** Statistical comparisons of alpha diversity (measured by the total number of observed species) of microbiota among the three sampling sources at 7010 sequences depths in *R. sinicus*.
